# Neutral Lipid Content in Lipid Droplets: Potential Biomarker of Cordycepin Accumulation in Cordycepin-Producing Fungi

**DOI:** 10.3390/molecules24183363

**Published:** 2019-09-16

**Authors:** Peng Qin, ZhiYe Wang, DengXue Lu, HongMei Kang, Guang Li, Rui Guo, YuHui Zhao, RongBing Han, Bing Ji, Yang Zeng

**Affiliations:** 1 Institute of Biology, Gansu Academy of Sciences, Lanzhou 730000, Gansu, China; p.qin@gsas.ac.cn (P.Q.); lzkanghm@163.com (H.K.); guoruihigh@163.com (R.G.); yuhuizhao51@163.com (Y.Z.); bingjie_@126.com (R.H.); jibingmountain@163.com (B.J.); zengyangfit@163.com (Y.Z.); 2 Key Laboratory of Microbial Resources Exploitation and Application of Gansu Province, Institute of Biology, Gansu Academy of Sciences, Lanzhou 730000, Gansu, China; 3 Analysis and Research Center of Gansu Province, Lanzhou 730000, Gansu, China; gsliguang@163.com

**Keywords:** lipid droplets, Nile red, cordycepin, *Cordyceps militaris*, mutants

## Abstract

To clarify the relationship between neutral lipid content and cordycepin accumulation in *Cordyceps* *militaris*, mutants were generated from mixed spores of two *C. militaris* strains with varying cordycepin-producing capacities. Fifteen stable mutants producing from 0.001 to 2.363 mg/mL cordycepin were finally selected. The relative fluorescence intensities of the 15 mutants, two *C. militaris* strains and an *Aspergillus nidulans* strain at different concentrations of lyophilized mycelium powder were then investigated using the Nile red method. The mutant CM1-1-1 with the highest relative fluorescence intensity among the eighteen strains was selected for optimizing the Nile red method. Relative fluorescence intensity was linearly correlated with cordycepin concentration in liquid broth (R^2^ = 0.9514) and in lyophilized mycelium powder (R^2^ = 0.9378) for the 18 cordycepin-producing strains under identical culture conditions and with cordycepin concentration in liquid broth (R^2^ = 0.9727) and in lyophilized mycelium powder (R^2^ = 0.9613) for CM1-1-1 under eight different sets of conditions. In addition, the cordycepin content in lyophilized mycelium powder measured by the Nile red method was linearly correlated with that determined by an HPLC method (R^2^ = 0.9627). In conclusion, neutral lipids in lipid droplets are required during cordycepin accumulation; these neutral lipids are potential biomarkers of cordycepin biosynthesis.

## 1. Introduction

Cordycepin (3′-deoxyadenosine), a cytotoxic nucleoside analogue found in *Cordyceps militaris*, has attracted considerable attention due to its diverse therapeutic potentials associated with anticancer, antitumor, anti-inflammatory, antimicrobial and antiviral effects [1,2,3]. Cordycepin serves as an important marker for the quality control of *C. militaris*.

The biosynthetic mechanism of cordycepin in purine biosynthesis has attracted widespread interest in the past seven decades since the discovery of cordycepin in 1950 [4]. Notably, based on a comparative genetic analysis of two distantly homogenous and cordycepin-producing fungi (*C. militaris* and *Aspergillus nidulans*), the Cns1 and Cns2 proteins, responsible for cordycepin biosynthesis, were determined to be colocalized on a type of organelle in *Cordyceps militaris* using GFP. Then, the Nile red method was employed to demonstrate that these organelles were lipid droplets in the mycelium of *C. militaris* [5]. To date, none of experiments have not been performed to investigate the effects of lipid droplets on Cns1 and Cns2 proteins in the mycelium of cordycepin-producing fungi. Previous work demonstrated that lipid droplets were involved in protein biosynthesis and degradation in eukaryotes [6], suggesting that the effects of lipid droplets on the Cns1 and Cns2 proteins in the mycelium of cordycepin-producing fungi were still unclear. For example, whether lipid droplets can modify Cns1 and Cns2 proteins in the mycelium of cordycepin-producing fungi requires further investigated. In other words, the lipid droplets in cells may have potential effects (positive or negative) on cordycepin accumulation in the mycelium of cordycepin-producing fungi.

Lipid droplets, spherical cellular organelles consisting of a neutral lipid core enclosed by a phospholipid monolayer membrane covered with many multifunctional binding proteins, are involved in multiple intracellular processes, such as protein storage and degradation and nucleic acid processing [6,7]. The neutral lipid content in cells is associated with lipid droplet content. The compositions of neutral lipids in lipid droplets markedly vary and are associated with cell type and culture conditions. Major types of neutral lipids in lipid droplets are different from those in fungi and mammals, such as triacylglycerols and steryl esters in fungi (budding yeast) [8], and triacylglycerol, cholesterol ester and ether lipids in in vivo mammalian cell lines [9]. However, to date, the quantitative relationship between the neutral lipid content and cordycepin accumulation in the mycelia of cordycepin-producing fungi remains unclear.

The relative fluorescence intensity of Nile red-stained smooth muscle cells was first used as a reliable indicator to quantify neutral lipid content [10], and the use of this proxy has been demonstrated in different microorganisms [11,12,13]. The strain, the solvent in which Nile red is dissolved and the measurement conditions affect the combination of Nile red and neutral lipid, causing variable relative fluorescence intensities [14]. Therefore, the measurement conditions for determining fluorescence intensity in a specific microorganism play a vital role in investigating the relationship between neutral lipid content and cordycepin accumulation in cordycepin-producing fungi.

In this study, fifteen mutant strains producing different levels of cordycepin were obtained from two *C. militaris* starting strains after LiCl+^60^Coγ compound mutagenesis. Lyophilized mycelium powder (LMP) was used as the original material to optimize the Nile red method to investigate the quantitative relationship between the cordycepin concentration in both liquid broth and LMP and the corresponding relative fluorescence intensity (RFI) from Nile red-stained LMP in cordycepin-producing fungi.

## 2. Results

### 2.1. Screening of Mutants with Varying Cordycepin Production

**Figure 1:**
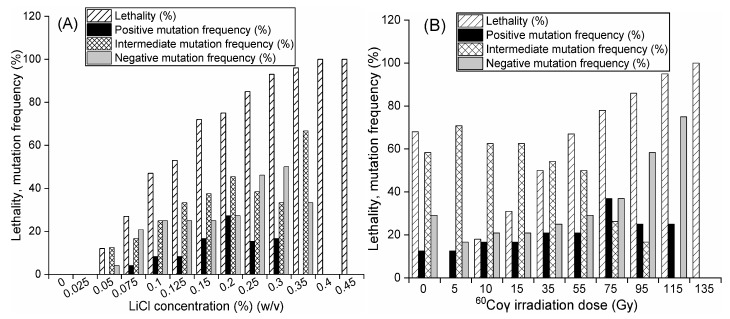
Lethality, positive mutant frequency, intermediate mutant frequency and negative mutant frequency in LiCl and LiCl+^60^Coγ mutagenesis. (**A**) LiCl mutagenesis. (**B**) LiCl+^60^Coγ compound mutagenesis. The results under 0 Gy irradiation were obtained by treating a mixed spore suspension with 0.2% LiCl for 12 h. The results from 5~135 Gy of irradiation were obtained by first treating mixed spore suspensions with 0.2% LiCl for 5 h and then exposing them to different doses of ^60^Coγ irradiation.

After exposure to different concentrations of LiCl, mutants were obtained from 12 different groups. The LiCl concentration was screened by lethality and mutation frequency (Figure 1A). According to the above indicators, 0.2% was selected as the optimal LiCl concentration, resulting in 75% lethality, 27% positive mutation frequency, 45% intermediate mutation frequency and 27% negative mutation frequency (Figure 1A).

**Figure 2:**
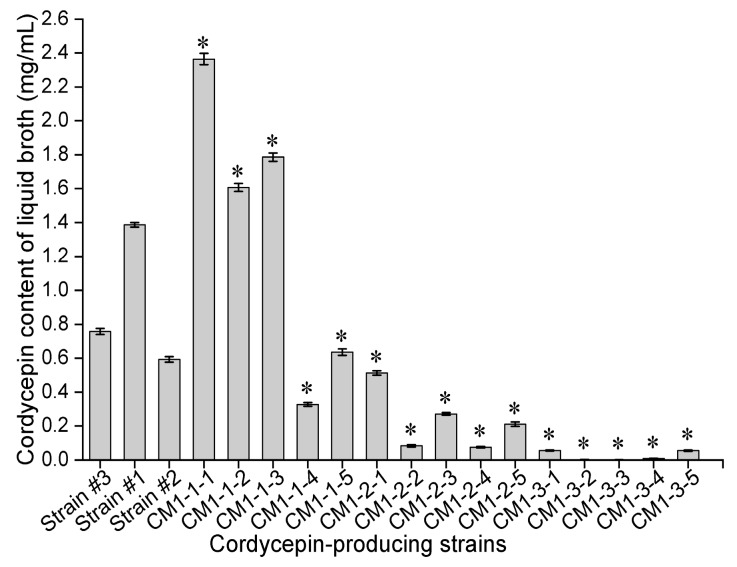
Cordycepin concentration in liquid broth of fifteen stable mutants, two *C. militaris* (strain #1 and strain #2) strains and an *A. nidulans* (strain #3) strain after 30 d of cultivation. The asterisks above each data were the results of statistical test (LSD). The cordycepin concentration in liquid broth of the 15 mutant strains was significantly different from that of the original strains (strain #1 and strain #2) (LSD, *p* < 0.05).

**Figure 3:**
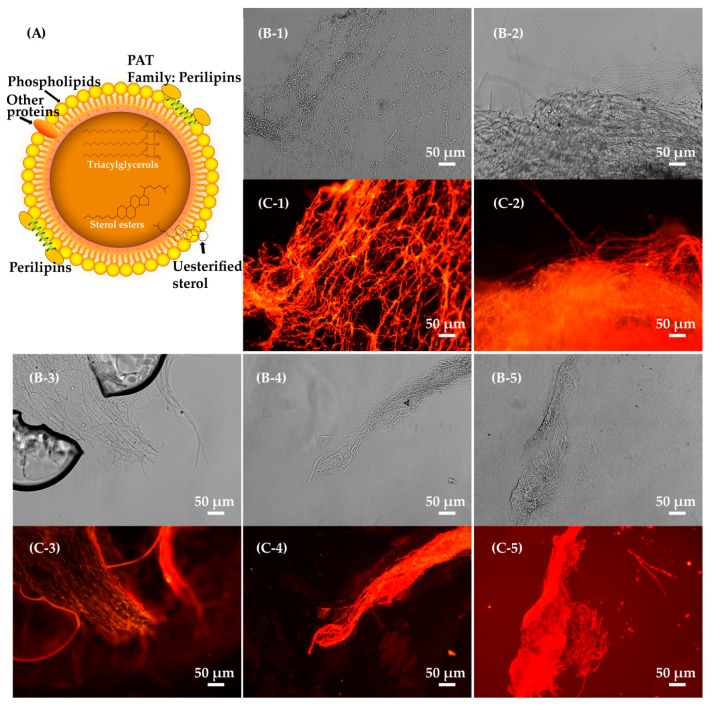
Image evidence of neutral lipids in lipid droplets in the mycelium of cordycepin-producing fungi. (**A**) Lipid droplet structure according to previous work [8,15]. The figure was generated by this work. (**B**) Mycelium stained with Nile red under the ordinary light of fluorescence microscope (BX35, OLYMPUS, Tokyo, Japan). (**C**) Mycelium stained with Nile red under the blue light (Wavelength 420−490 nm) of fluorescence microscope (BX35, OLYMPUS, Tokyo, Japan). Note: Figure B (C)-1, B (C)-2, B (C)-3, B (C)-4 and B (C)-5 represent CM1-1-1, strain #1, strain #3, strain #2 and CM1-3-3, respectively. Each strain was inoculated on PDA slant, cultured at 25 °C for 9 d. Then, mycelium of each strain were collected, washed with PBS thrice, treated with Nile red solution (Nile red/DMSO) at a final concentration of 1 µg/mL for 5 min, washed with PBS once, observed under the ordinary light and the blue light (wavelength 420–490 nm), respectively. Cell membrane lipids of mycelium emitted red fluorescence under the blue light. Neutral lipids in lipid droplets in the mycelium stained with Nile red emitted yellow fluorescence under the blue light. The microscope magnificationis 200×.

After mutagenesis with the optimal concentration of LiCl and subsequent exposure to different doses of ^60^Coγ irradiation, ten groups of mutants were obtained from the mutant mixed spore suspensions (MSSs) (Figure 1B). The lethality and mutation frequency were measured by a lethality assay and antimicrobial tests. Mutants (treated with 0–135 Gy ^60^Coγ irradiation) were preliminarily screened according to the inhibition diameter of each mutant in antimicrobial tests (Figure 1B). Fifteen stable mutants that had positive (named CM1-1-(1–5)), intermediate (named CM1-2-(1–5)) and negative (named CM1-3-(1–5)) inhibition diameters were finally selected after six generations of short-term cultivation. After 30 d of static cultivation at 25 °C in darkness, the cordycepin concentrations in liquid broth of the fifteen stable mutants and three wild strains (strain #1, strain #2 and strain #3) were determined using high-performance liquid chromatography (HPLC). As shown in Figure 2, the cordycepin concentrations in liquid broth of these eighteen strains varied from 0.001 (CM1-3-3) to 2.363 (CM1-1-1). As shown in Figure 3, the intensities of yellow fluorescence, emitted by neutral lipids in lipid droplets in the mycelium of CM1-1-1, strain #1, strain #3, strain #2 and CM1-3-3 stained with Nile red, decreased with a respective decrease of cordycepin concentration in liquid broth, which indicated a potential relationship between cordycepin accumulation and neutral lipid content in the mycelium of cordycepin-producing fungi. Then, the cordycepin concentration in liquid broth and cordycepin content in LMP of these eighteen strains (strain #1~strain #3 and CM1-1-1~CM1-3-5) were used to quantify the cordycepin concentration in liquid broth and cordycepin content in LMP by the Nile red method.

### 2.2. Fluorescence Characteristics of Mutant CM1-1-1 Stained with Nile Red

Under a multiscan spectrum with an excitation wavelength of 490 nm and emission wavelength of 550–650 nm, RFI from Nile red-stained LMP of the mutant CM1-1-1 was markedly distinguished from that of unstained LMP and that of Nile red, showing a similar emission peak at 598 nm. The fluorescence intensities from Nile red and unstained LMP were rather low. Therefore, a 490 nm excitation wavelength and a 598 nm emission wavelength were applied in the following experiments.

### 2.3. Fluorescence Intensities of 18 Selected Strains Stained with Nile Red

**Figure 4:**
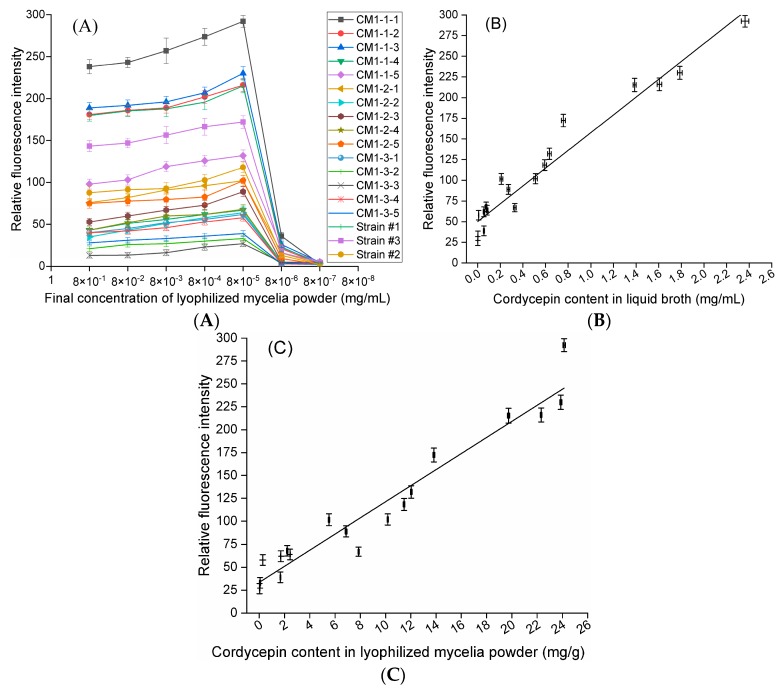
Relative fluorescence intensities from Nile red-stained lyophilized mycelium powder of six strains and corresponding cordycepin concentration in liquid broth. (**A**) Relative fluorescence intensities from 18 strains at different concentrations. (**B**) The relative fluorescence intensities of 8 × 10^−5^ mg/mL lyophilized mycelium powder of 18 strains are linearly correlated with the corresponding cordycepin concentration in liquid broth (R^2^ = 0.9514). (**C**) The relative fluorescence intensities of 8 × 10^−5^ mg/mL lyophilized mycelium powder of 18 strains are linearly correlated with the corresponding cordycepin content in lyophilized mycelium powder (R^2^ = 0.9378).

The effects of different LMP concentrations of the 18 selected strains on the RFI were investigated (Figure 4A). The RFIs of the Nile red-stained LMPs were distinguished but exhibited similar characteristics: Each slightly increased with increasing final LMP concentration, reached a peak when the final LMP concentration was 8 × 10^−5^ mg/mL, and rapidly decreased when the final LMP concentration was greater than 8 × 10^−5^ mg/mL, indicating that a final concentration of at most 8 × 10^−5^ mg/mL LMP should be used when the final concentration of Nile red reached 3.2 µg/mL (Figure 4A). Notably, the RFI from Nile red-stained LMP was linearly correlated with the corresponding cordycepin concentration in liquid broth (R^2^ = 0.9514) (Figure 4B) and cordycepin content in LMP (R^2^ = 0.9378) (Figure 4C) when the final LMP concentration was 8 × 10^−5^ mg/mL. Therefore, the mutant CM1-1-1, with the highest RFI (292), cordycepin concentration in liquid broth (2363.278 µg/mL) and cordycepin content in LMP (24.14 mg/g) among the 18 strains, was selected for subsequent experiments.

### 2.4. Optimizing Measurement Conditions for the Nile Red Method Using Mutant CM1-1-1

**Figure 5:**
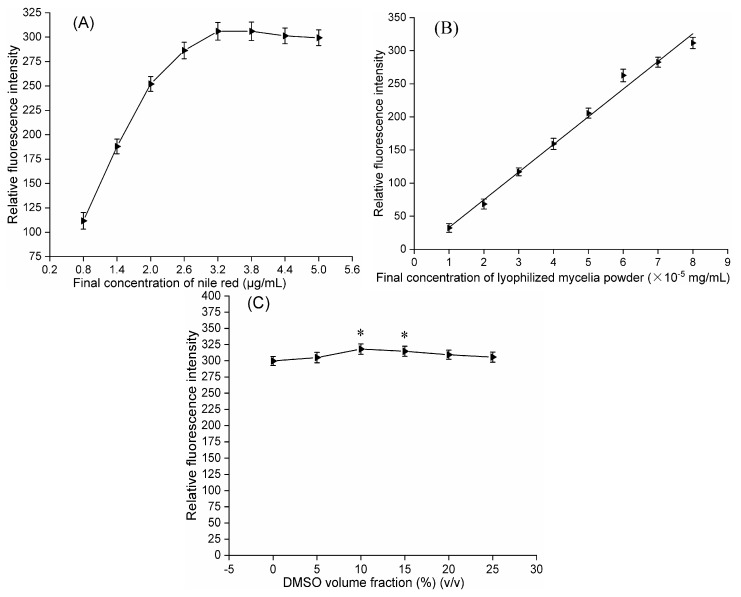
Optimizing conditions for determining the relative fluorescence intensity from mutant CM1-1-1. (**A**) Response of relative fluorescence intensity to different concentrations of Nile red. (**B**) Effect of different concentrations of lyophilized mycelium powder on fluorescence intensity. (**C**) Effect of pretreatment with different volume fractions of DMSO on relative fluorescence intensity. The asterisks above data points indicate a significant difference from the control (LSD, *p* < 0.05).

The effect of different final concentrations of Nile red (0.8, 1.4, 2.0, 2.6, 3.2 and 3.8 µg/mL) on the RFI from Nile red-stained LMP of mutant CM1-1-1 was investigated. Figure 5A shows that the RFI rapidly increased when the Nile red concentration was less than 3.2 µg/mL, consistently peaked within the Nile red range of 3.2–3.8 µg/mL and slightly decreased when the Nile red concentration was greater than 3.8 µg/mL. Therefore, at least 3.2 µg/mL Nile red is needed when the LMP final concentration is 8 × 10^−5^ mg/mL.

The effects of different final concentrations of LMP (1 × 10^−5^, 2 × 10^−5^, 3 × 10^−5^, 4 × 10^−5^, 5 × 10^−5^, 6 × 10^−5^, 7 × 10^−5^ and 8 × 10^−5^ mg/mL) on the RFI from Nile red-stained LMP of mutant CM1-1-1 were investigated by mixing 120 µL of LMP suspension at different concentrations with 30 µL of Nile red solution to a final concentration of 3.2 µg/mL. Figure 5B shows that the RFI was linearly correlated with the final LMP concentration from 1 × 10^−5^~8 × 10^−5^ mg/mL, which was quantitatively expressed as y = 41.905x − 8.571 (y: RFI; x: final concentration of LMP × 10^−5^ mg/mL; R^2^ = 0.9906).

The effect of different volume fractions of dimethyl sulfoxide (DMSO) (5, 10, 15, 20 and 25%) (*v*/*v*) on the RFI from Nile red-stained LMP of mutant CM1-1-1 was investigated (Figure 5C). To maintain the optimal final LMP concentration at 8 × 10^−5^ mg/mL, each LMP suspension in phosphate-buffered saline (PBS) (1.05 × 10^−4^, 1.11 × 10^−4^, 1.18 × 10^−4^, 1.25 × 10^−4^ and 1.33 × 10^−4^ mg/mL) was individually pretreated with DMSO at different volume fractions (LMP suspension: DMSO = 19:1, 9:1, 17:3, 4:1 and 3:1) before fluorescence intensities were determined. The RFI slowly increased with increasing DMSO volume fraction, peaked when the DMSO volume fraction was 10% and slightly decreased when the DMSO volume fraction was greater than 10% (Figure 5C). Therefore, 10% DMSO was suitable for enhancing RFI.

### 2.5. Quantification of Cordycepin Concentration of Liquid Broth and Cordycepin Content in LMP by the Relative Fluorescence Intensity from Mutant CM1-1-1

**Figure 6:**
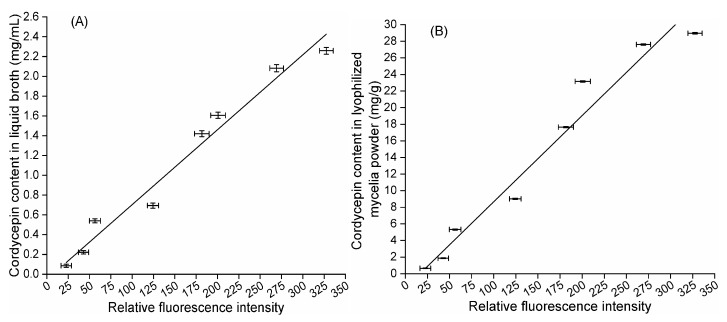
Quantitative correlation between cordycepin content and relative fluorescence intensity from Nile red-stained lyophilized mycelium powder of mutant CM1-1-1. (**A**) The relationship between cordycepin concentration in liquid broth and relative fluorescence intensity from Nile red-stained lyophilized mycelium powder. (**B**) The relationship between cordycepin content in lyophilized mycelium powder and relative fluorescence intensity from Nile red-stained lyophilized mycelia powder.

Eight samples of LMP harvested under different culture conditions (#1–#8) were individually diluted in PBS (1.11 × 10^−4^ mg/mL), mixed with 10% (*v*:*v*) DMSO to 1 × 10^−4^ mg/mL, and reacted in darkness at 30 °C for 10 min. Then, 120 μL of LMP suspension was mixed with 30 µL of Nile red solution at a final concentration of 3.2 µg/mL and thoroughly vortexed for 5 min at 30 °C prior to the determination of fluorescence intensities. Figure 6A shows that the cordycepin concentration of liquid broth under eight different conditions was linearly correlated with the corresponding RFI from Nile red-stained LMP, and the relationship can be described by y = 0.007x − 0.029 (y: cordycepin concentration of liquid broth; x: RFI from Nile red-stained LMP; R^2^ = 0.9727). Figure 6B shows that the cordycepin content in LMP under eight different conditions was linearly correlated with the corresponding RFI from Nile red-stained LMP, which can be quantified as y = 0.102x − 1.311 (y: cordycepin content in LMP; x: RFI from Nile red-stained LMP; R^2^ = 0.9613).

### 2.6. The Relationship between Cordycepin Content in LMP Measured by HPLC and that Measured by Fluorescence

**Figure 7:**
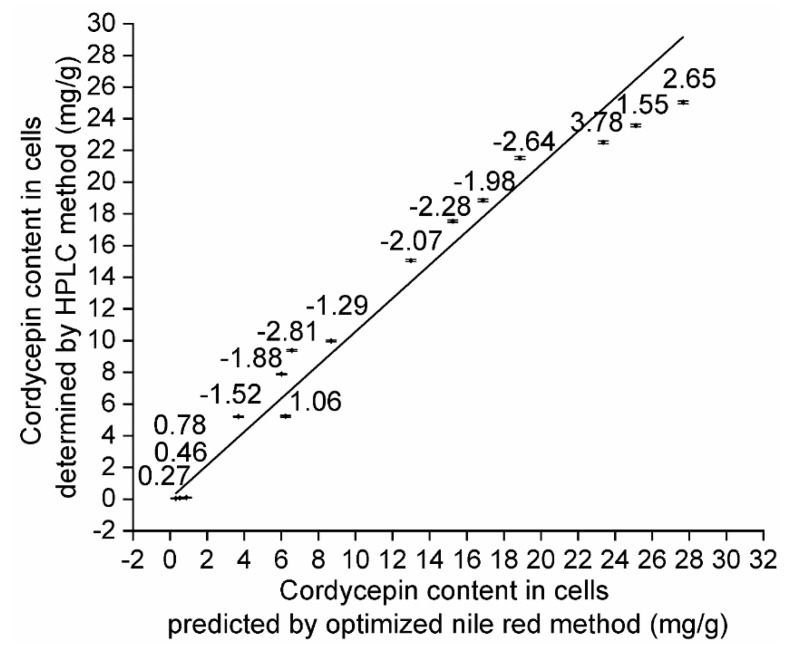
The relationship between cordycepin content in LMP determined by HPLC and that measured by the optimal Nile red method. The data near each point is the absolute error of cordycepin content in LMP individually measured by two methods, and the absolute error is expressed as y = a − b (y: absolute error; a: predicted value measured by the optimal Nile red method; b: true value determined by HPLC method). The relative error of all samples is less than 6%, which can be expressed as y = ((a − b) ÷ b) × 100% (y: relative error; a: predicted value measured by the optimal Nile red method; b: true value, determined by HPLC method).

Single colonies of strain #1, strain #2, CM1-1-1, CM1-2-5 and CM1-3-3, prepared using the method described in Section 4.3, were individually and statically cultured at 25 °C in 20 mL basic liquid medium per 50 mL centrifuge tube for different culture times (20, 25 and 30 d). Each experiment was performed in triplicate. LMP was prepared according to the method in Section 4.5, and the fluorescence intensities of LMP were determined by the optimal Nile red method as in Section 2.5. Cordycepin content in LMP, the same as in cells, was calculated using quantitative equation y = 0.102x − 1.311 as in Section 2.5 and determined by HPLC method as in Section 4.4 and Section 4.5. As shown in Figure 7, the cordycepin content in LMP predicted by the optimized Nile red method was linearly correlated with that determined by the HPLC method (R^2^ = 0.9627).

## 3. Discussion

In our study, an optimized methylcellulose-based medium, a type of semisolid medium, was first used for isolating mutant spores of fungi. In brief, a fungal spore suspension was piped into optimized semisolid methylcellulose medium, and the medium thoroughly shaken to homogeneously disperse these spores. Then, the relative positions of these spores in optimized semisolid methylcellulose medium were fixed during static cultivation, and these spores formed single colonies and were separately transferred into downstream medium in 96-deep-well plates using a pipette with a 1000 μL tip. Notably, compared to traditional methods for isolating single fungal spores, such as the spread plate method, this novel method is simple, convenient and efficient for several reasons: (a) optimized semisolid methylcellulose medium is liquid at 0~70 °C, which is suitable for the growth of fungi; (b) pipette operation is simple, convenient and efficient; and (c) single spores can form single colonies inside the medium and on the surface of the medium. In addition, LMP of fungi was first used as the original material to measure neutral lipid content in lipid droplets using the Nile red method, and the quantitative relationships between the cordycepin concentration in liquid broth, cordycepin content in LMP, and corresponding RFI from Nile red-stained LMP were investigated. Eighteen stable mutants (CM1-1-1~CM1-3-5), individually producing from 0.001 (CM1-3-3) to 2.363 (CM1-1-1) mg/mL cordycepin, were finally obtained after LiCl+^60^Coγ compound mutagenesis. The RFI values from the Nile red-stained LMP of these cordycepin-producing strains were linearly correlated with the corresponding cordycepin concentration in liquid broth (R^2^ = 0.9332) when the LMP final concentration was 8 × 10^−5^ mg/mL. The mutant CM1-1-1, with the highest RFI from Nile red-stained LMP among the eighteen strains, was selected for optimizing the Nile red method, and the optimal method for RFI determination can be described as follows: 120 μL of LMP suspension, which was obtained by mixing LMP suspension prepared in PBS (1.11 × 10^−4^ mg/mL) with 10% DMSO (*v*/*v*) prior to a reaction in darkness at 30 °C for 10 min, was mixed with 30 µL of Nile red solution at final concentration of 3.2 µg/mL and thoroughly vortexed for 5 min at 30 °C prior to the determination of fluorescence intensities. Using this optimal method, the cordycepin concentration in liquid broth (R^2^ = 0.9727) and cordycepin content in LMP (R^2^ = 0.9613) were demonstrated to be linearly correlated with the corresponding RFI from Nile red-stained LMP of eight samples of mutant CM1-1-1 harvested under different culture conditions. In addition, the cordycepin content in LMP measured by the optimized Nile red method was linearly correlated to that determined by an HPLC method using five strains cultured for different times (R^2^ = 0.9627). In conclusion, in 18 cordycepin-producing fungi, neutral lipids in lipid droplets are required during cordycepin accumulation and considered potential biomarkers of cordycepin biosynthesis.

Cordycepin is a self-toxic component in cordycepin-producing fungi. These fungi can develop the capacity to resist and detoxify this toxic compound. 3′AMP is potentially formed and transferred into lipid droplets, and then cordycepin is biosynthesized by the two enzymes Cns1 and Cns2, which are colocalized on lipid droplets [5]. Our data showed a significant relationship between the RFI from Nile red-stained LMP and cordycepin accumulation in cordycepin-producing fungi, suggesting a marked correlation between neutral lipids in lipid droplets and cordycepin biosynthesis. Therefore, we speculate that lipid droplets in these cordycepin-producing fungi enclose cordycepin to prevent it from contacting targets within their own cell and transport cordycepin to the vicinity of the cell membrane. Then, cordycepin is pumped into the extracellular matrix by a specific transporter. This mechanism potentially enhances the resistance and detoxification capacities of these fungi toward self-toxic cordycepin. In other words, lipid droplets may improve the cordycepin-producing capacity of these fungi. Thus, at most, 25.93% cordycepin remained in the cells of these strains (cultured at 25 °C for 30 d) used in our experiments. In other words, over 74.07% of cordycepin was pumped into liquid broth. Among these strains, mutant CM1-1-1 retained the least cordycepin in cells, which was less than 12.6%, under different culture conditions. In brief, neutral lipids in lipid droplets in the mycelium of cordycepin-producing fungi may be associated with detoxification of toxic cordycepin, but the effects are limited.

In addition, the multiple functions of lipid droplet-associated proteins may illuminate the positive effect of lipid droplets on cordycepin accumulation. Since lipid droplet-associated proteins vary substantially in different fungi, lipid droplet-resident proteins are involved in different signals, such as those involving signal peptides, posttranslation modification and nucleic acids, which may help these proteins localize lipid droplets [6]. Notably, previous work demonstrated that *C. militaris* mutant G81-3 produced abundant cordycepin in the mycelial stage by liquid surface culture. The over saturation of cordycepin resulted in crystallization when the cordycepin concentration in liquid broth exceeded approximately 7.4 g/L at 25 °C. The maximum capacity of cordycepin production reached 14.3 g/L through a decrease in the beginning substrate level and a water-supplementing strategy [16]. Then, some work for the strain G81-3 in the mycelia stage when grown in liquid surface culture need further investigation to take better advantage of this excellent strain: (a) Whether cordycepin crystallization occurs in cells; (b) if cordycepin crystallization appears in cells, how do G81-3 cells respond to this issue; and (c) if cordycepin is biosynthesized without crystallization in cells throughout the mycelial stage, in spite of cordycepin being pumped outside cells through cordycepin transporters, can additional mechanisms also decrease the effective concentration of cordycepin in cells? For example, cordycepin might be enclosed in neutral lipids in lipid droplets of cells to downregulate the cordycepin level in the cytoplasm. (d) the detailed mechanisms that G81-3 can produced such large amount of cordycepin in the mycelia stage require further investigation. In addition, abundant cordycepin is produced during the fruiting stage of *C. militaris*; during this time, signal transduction is markedly more active than it is in mycelia [17]. As a result, the neutral lipid content may increase to overcome the unfavourable increase in cordycepin during the fruiting stage of cordycepin-producing fungi. Therefore, more cordycepin may be produced by mutant G81-3 in the fruiting stage than in the mycelial stage if G81-3 can form a fruiting body. Further investigations are required to clarify these issues. Our future work will focus on analysing the genetic expression and dynamics involved in droplet synthesis and cordycepin accumulation in *C. militaris* cells.

## 4. Materials and Methods

### 4.1. Strains and Medium

*C. militaris* CGMCC 3.4655 (strain#2), *Bacillus subtilis* CGMCC 1.1849 and *A. nidulans* CGMCC 3.15737 (strain#3) were obtained from the China General Microbiological Culture Collection Center (Beijing, China). *C. militaris* CICC 14014 (strain #1) was obtained from the China Center of Industrial Culture Collection (Beijing, China). *C. militaris* and *A. nidulans* were maintained on PDA and YPD slants, respectively. *Bacillus subtilis* was cultured on nutrient agar (NA) medium. Basic liquid medium (g/L): glucose, 34; peptone, 17; KH_2_PO_4_, 1; and MgSO_4_·7H_2_O, 1. Optimized semisolid medium (g/L): basic liquid medium; methylcellulose, 23. Medium #A (g/L): glucose, 16; peptone, 7; KH_2_PO_4_, 1; and MgSO_4_·7H_2_O, 1. Medium #B (g/L): glucose, 6; peptone, 2; KH_2_PO_4_, 1; and MgSO_4_·7H_2_O, 1.

### 4.2. Materials

Nile red, DMSO and methylcellulose (viscosity: 4000 MPa·s) were obtained from Shanghai Sangon Biotech Limited Corporation (Shanghai, China). Nile red solution: 0.1 mg per 1 mL of acetone, was diluted with acetone to the appropriate concentration just prior to each assay. Cordycepin was obtained from the National Institutes for Food and Drug Control (Beijing, China). LiCl was obtained from Sinopharm Chemical Reagent Limited Corporation (Beijing, China). Methylene blue solution (0.1%) was obtained from Beijing Solarbio Science and Technology Co., Ltd. (Beijing, China).

### 4.3. Preparation of Spore Suspension, Lethality Assays and Antimicrobial Tests

Spore suspensions of strain #1 and strain #2, which had markedly different cordycepin-producing capabilities, were individually diluted with sterile PBS (pH 7.2) to 1 × 10^6^ living conidia/mL and examined by methylene blue staining, optical microscopy (CX23, OLYMPUS, Tokyo, Japan) and hemocytometry. MSSs of strain #1 and strain #2 were prepared by mixing the same volume of each suspension.

Mutant MSSs were appropriately diluted with sterile PBS, transferred into 20 mL of optimized semisolid medium in a 50 mL Erlenmeyer flask, fully shaken to homogeneously blend the spores, and incubated statically in darkness at 25 °C for 72 h. The relative positions of mutant MSSs in optimized semisolid medium were fixed during static cultivation. Variable isolates were transferred into downstream medium using a pipette with a 1000 µL tip. Lethality was calculated with the equation below by observing different isolates in optimized semisolid medium.

Eight random isolates under each mutation condition were separately transferred into 1.8 mL of basic liquid medium per well in 96-deep-well plates and incubated statically at 25 °C in darkness for 10 d (LiCl mutation) or 30 d (LiCl+^60^Coγ compound mutagenesis and screening of stable mutants). Antimicrobial tests were performed with *B. subtilis* [18]. Eight sterile paper disks (7 mm) saturated with liquid broth were placed on each NA plate and incubated in darkness at 30 °C for 12 h. Lethality and mutant frequency were calculated by comparing these groups with the nonirradiated groups:

(1) $$\mathrm{Lethality}\left(\%\right)=\frac{\mathrm{NB}-\mathrm{NA}}{\mathrm{NB}}\times 100,$$

(2) $$\mathrm{PMF}\left(\%\right)=\frac{\mathrm{NEC}}{\mathrm{NVC}}\times 100,$$

(3) $$\mathrm{NMF}\left(\%\right)=\frac{\mathrm{NDC}}{\mathrm{NVC}}\times 100,$$

(4) $$\mathrm{IMF}\left(\%\right)=\left(1-\frac{\mathrm{PMF}+\mathrm{NMF}}{100}\right)\times 100$$

Using Equations (1)–(4) the mutants which showed an inhibition diameter over 20% larger than that of strain #1 were defined as positive mutants, and the mutants with a 20% or smaller inhibition diameter than strain #2 were considered negative mutants. NB is the number of single colonies before mutagenesis, NA is the number of single colonies after mutagenesis, PMF is the positive mutant frequency, NEC is the number of positive mutant colonies, NVC is the number of viable colonies, NMF is the negative mutant frequency, IMF is the intermediate mutant frequency and NDC is the number of negative mutant colonies.

### 4.4. Mutation Procedure

According to the modified mutation method using LiCl [19] that stimulated base substitution and significantly enhanced the mutation efficiency in combined mutagenesis [19,20], MSSs were incubated with different concentrations of LiCl (0.025, 0.05, 0.075, 0.1, 0.125, 0.15, 0.2, 0.25, 0.3, 0.35, 0.4 and 0.45%) (*w*/*v*) prepared in sterile PBS and cultured in darkness at 25 °C for 12 h. Lethality assays and antimicrobial tests were performed, and the optimal concentration of LiCl was selected according to lethality and mutant frequency.

According to the modified mutation method under ^60^Coγ ray [21], which mechanistically induced a large number of double-stranded breaks of chromosomal DNA and caused genetic variability [22], after treatment with LiCl at the optimal concentration for 5 h, MSSs were irradiated under a ^60^Coγ ray (^60^Co irradiator, CN-101, CNNC, Gansu Tianchen Irradiation Technology Co. Ltd., Lanzhou, China) at different doses (5, 10, 15, 35, 55, 75, 95, 115 and 135 Gy) for 15 min. Positive, intermediate and negative mutants under each dose of ^60^Coγ irradiation were preliminarily screened according to their inhibition diameters being obviously different from those of their initial strains in antimicrobial tests. Throughout six d of cultivation on PDA slants for six generations, 15 stable mutants were individually cultured at 25 °C for 30 d, selected by comparing the inhibition diameters of liquid broth obtained from mutants of the first generation with those of the corresponding mutants of the sixth generation using antimicrobial test method as described in Section 4.3.

A single colony of each selected strain (stable mutants, strain #1, strain #2 and strain #3), prepared using the method described in Section 4.3, was individually transferred into 20 mL of basic liquid medium in a 50 mL centrifuge tube and incubated in darkness at 25 °C for 30 d, and liquid broth was filtered through 22 µm filter paper prior to determining the cordycepin concentration in liquid broth using HPLC. HPLC was performed with the following parameters: Agilent 1260 platform (Santa Clara, CA, USA), DiamonsilC18(2) (5 μm × 4.6 mm × 250 mm), 15% methanol (Merck, Germany): 85% water (*v*/*v*), flow rate: 1 mL/min, 35°C, injection volume: 20 µL, and detection wavelength: 260 nm. The mycelia of these strains were used for determining fluorescence intensities.

### 4.5. LMP Preparation

The mycelia of the stable mutants, strain #1, strain #2 and strain #3 were filtered with three-layer gauze, washed with pure water three times, frozen in a cryogenic refrigerator (Forma 900 series, Thermo, Waltham, MA, USA) at −80 °C for 12 h, dried in a vacuum freeze dryer (Labconco, Kansas, MO, USA) for 28 h, ground into LMP in a mortar, and then filtered through a 90 mesh sieve. The mortar and sieve were washed with 95% ethanol three times prior to the next LMP being treated. According to a previously described method [23], water extracts (10 mL) of treated LMP (0.1 g) of the stable mutants, strain #1, strain #2 and strain #3 were prepared to determine the cordycepin concentration in the cells using HPLC. The residual treated LMP was stored at −20 °C before being used in subsequent experiments.

### 4.6. Determination of Fluorescence Intensity by a Multiscan Spectrum

LMP (10 ± 0.1 mg) was weighed with an electronic balance (Shanghai Yueping Scientific Instrument Co., Ltd., Shanghai, China) and diluted with 10 mL of PBS prior to each assay. Then, 120 µL of LMP suspension was mixed with 30 μL of Nile red solution at a final concentration of 3.2 μg/mL. Treated LMP was agitated on a 96-well plate oscillator vigorously for 5 min before determining the fluorescence intensities on a microplate reader (Infinite M200 pro, Männedorf, Kanton Zürich, Switzerland) with a 490 nm excitation wavelength and a 598 nm emission wavelength. The RFI was calculated after subtracting the self-fluorescence of Nile red and the fluorescence intensity of the LMP suspension at 598 nm.

### 4.7. Optimizing the Nile red Method

For optimization, 120 µL of LMP suspensions at seven different concentrations (1, 1 × 10^−1^, 1 × 10^−2^, 1 × 10^−3^, 1 × 10^−4^, 1 × 10^−5^ and 1 × 10^−6^ mg/mL) were mixed with 30 µL of Nile red solution at a final concentration of 3.2 µg/mL and vortexed vigorously in darkness at 30 °C for 5 min [24] before determining the fluorescence intensities. The relationships between the cordycepin concentration in liquid broth, cordycepin concentration in LMP, and the corresponding RFI from Nile red-stained LMP were evaluated at the optimal concentration of each LMP for the selected eighteen strains, with the highest RFI from Nile red-stained LMP determined at seven different concentrations. The strain with the maximal RFI from Nile red-stained LMP at the optimal concentration was selected for the following experiments.

Next, 120 µL aliquots of LMP suspension of the selected strain at the optimal concentration were individually mixed with 30 µL of different Nile red solutions in acetone (4, 7, 10, 13, 16 and 19 µg/mL) at different final concentrations (0.8, 1.4, 2.0, 2.6, 3.2 and 3.8 µg/mL) and vortexed vigorously at 30°C in darkness for 5 min prior to determining the fluorescence intensity.

Then, 120 µL of LMP suspensions of the selected strain at eight different concentrations (1, 0.875, 0.75, 0.625, 0.5, 0.375, 0.25 and 0.125 × 10^−4^ mg/mL) were individually mixed with 30 µL of Nile red solution at the optimal final concentration and vortexed vigorously at 30 °C in darkness for 5 min before the determination of the fluorescence intensity.

To maintain the optimal final concentration of LMP, LMP suspensions were individually mixed with a volume ratio of DMSO (5%, 10%, 15%, 20% and 25%) (*v*/*v*) and reacted in darkness at 30 °C for 10 min. For this purpose, 120 µL of pretreated LMP suspension was mixed with 30 µL of Nile red solution at the optimal final concentration and vortexed vigorously in darkness at 30 °C for 5 min before determining fluorescence intensity.

### 4.8. Cordycepin Content Quantification by Relative Fluorescence Intensity

A single colony of the selected strain that was prepared using the method described in Section 4.3 was transferred into 20 mL of basic liquid medium in a 50 mL centrifuge tube and statically cultured under eight different conditions (#1–#8) as follows: conditions #1–#2 consisted of 25°C, 30 d and different media (medium #A and medium #B), while conditions #3–#8 consisted of 25°C, different culture times (5, 10, 15, 20, 25 and 30 d) and basic liquid medium. The relationships between the cordycepin concentration in liquid broth, cordycepin content in LMP and the corresponding RFI from Nile red-stained LMP were investigated.

### 4.9. Data Analysis

Three replicates of each experiment were performed. The results were expressed as the mean ± standard deviation and analysed through one-way ANOVA using SPSS 22.0 software (International Business Machines Corporation, Armonk, NY, USA). Statistical differences at the 0.05 level using the least significant difference (LSD) method were considered significant.
